# Supplementary material for: Combined effect of the entomopathogenic fungus Metarhizium robertsii and avermectins on the survival and immune response of Aedes aegypti larvae
Source: PeerJ. 2019 Oct 25;7:e7931. doi: 10.7717/peerj.7931 (PMC6816395; doi:10.7717/peerj.7931)
Supplement: Table S1 — *, Significant synergistic effects (χ2 > 18.5, df = 1, P < 0.001) calculated as described by Robertson and Preisler (1992) [file peerj-07-7931-s002.docx]

|  | Days posttreatment | | | | | |
| --- | --- | --- | --- | --- | --- | --- |
|  | 1 | 2 | 3 | 4 | 5 | 6 |
| Observed mortality | 3.3 | 32.0 | 66.0 | 85.3 | 92.7 | 98.7 |
| Expected mortality | 0 | 18.4 | 37.1 | 54.2 | 65.3 | 80.8 |
| Observed–expected | 3.3 | 13.6 | 28.9 | 31.1 | 27.4 | 17.8 |
| Chi square | – | 18.5* | 53.8* | 58.5* | 49.6* | 30.8* |
